# *R*otational Thrombo*E*lastometry-guided blood component administration versus standard of care in patients with *C*irrhosis and coagulopathy undergoing *I*nvasive *P*roc*E*dures (RECIPE): study protocol for a randomised controlled trial

**DOI:** 10.1186/s13063-023-07552-1

**Published:** 2023-08-11

**Authors:** Natasha Janko, Ammar Majeed, William Kemp, Chris Hogan, Harshal Nandurkar, Stuart K. Roberts

**Affiliations:** 1 Department of Gastroenterology, Alfred Health, Melbourne, VIC Australia; 2 Central Clinical School, Monash University, Melbourne, VIC Australia; 3 Department of Laboratory Haematology, Austin Health, Melbourne, VIC Australia; 4 Department of Haematology, Alfred Health, Melbourne, VIC Australia; 5 Australian Centre for Blood Diseases, Monash University, Melbourne, VIC Australia

**Keywords:** Rotational Thromboelastometry (ROTEM), Viscoelastic tests (VETs), Chronic liver disease, Coagulopathy, Surgery, Cirrhosis, Transfusion, Fresh frozen plasma, Platelets

## Abstract

**Background:**

Patients with cirrhosis often undergo invasive procedures both for management of complications of their advanced liver disease, including treatment for hepatocellular carcinoma, as well as underlying comorbidities. Despite a current understanding that most patients with cirrhosis are in a rebalanced haemostatic state (despite abnormalities in conventional coagulation tests, namely INR and platelet count), patients with cirrhosis are still often given prophylactic blood components based on these conventional parameters, in an effort to reduce procedure-related bleeding. Viscoelastic tests such as Rotational Thromboelastometry (ROTEM) provide a global measurement of haemostasis and have been shown to predict bleeding risk more accurately than conventional coagulation tests, and better guide blood product transfusion in a number of surgical and trauma-related settings. The aim of this study is to assess the utility of a ROTEM-based algorithm to guide prophylactic blood component delivery in patients with cirrhosis undergoing invasive procedures. We hypothesise that ROTEM-based decision-making will lead to a reduction in pre-procedural blood component usage, particularly fresh frozen plasma (FFP), compared with standard of care, whilst maintaining optimal clinical outcomes.

**Methods:**

This is a multi-centre randomised controlled trial comparing ROTEM-guided prophylactic blood component administration to standard of care in patients with cirrhosis and coagulopathy undergoing invasive procedures. The primary efficacy outcome of the trial is the proportion of procedures requiring prophylactic transfusion, with the primary safety outcome being procedure-related bleeding complications. Secondary outcomes include the amount of blood products (FFP, platelets, cryoprecipitate) transfused, transfusion-related side effects, procedure-related complications other than bleeding, hospital length of stay and survival.

**Discussion:**

We anticipate that this project will lead to improved prognostication of patients with cirrhosis, in terms of their peri-procedural bleeding risk. We hope to show that a significant proportion of cirrhotic patients, deemed coagulopathic on the basis of standard coagulation tests such as INR and platelet count, are actually in a haemostatic balance and thus do not require prophylactic blood product, leading to decreased and more efficient blood component use.

**Trial registration:**

RECIPE has been prospectively registered with the Australia and New Zealand Clinical Trials Registry on the 30th April 2019 (ACTRN12619000644167).

## Introduction

### Background and rationale {6a}

The peri-procedural assessment of haemostasis in patients with cirrhosis remains challenging for clinicians.

Historically, cirrhosis had been labelled as a bleeding disorder due to the abnormalities in conventional coagulation tests seen, namely prolonged prothrombin time (PT)/international normalised ratio (INR), and thrombocytopenia. On this basis, traditionally, attempts have been made to correct these parameters with blood component prophylaxis prior to invasive procedures in this patient population [1].

More recently, the concept of “rebalanced haemostasis” in cirrhosis was developed and is now well-established [2]. In addition to the reduction in procoagulants, and thrombocytopenia typically associated with advanced liver disease, there is also a reduction in anticoagulants and an increase in von Willebrand factor and factor VIII [3], leading to a new haemostatic balance. This balance, however, is delicate and can be easily disturbed, predisposing the patient with cirrhosis to both bleeding and thrombotic complications [4].

While patients with cirrhosis do have an increased risk of bleeding complications, aside from portal hypertensive-related events, they experience disproportionately fewer bleeding complications than would be expected by their INR/PT and platelet count values [5]. For most patients, the increased bleeding risk relates to portal hypertension, endothelial dysfunction, bacterial infection, and renal failure rather than defective haemostasis (at least that which is assessed by INR/platelet count) [6–8]. There is now robust evidence that INR/PT do not predict procedure-related bleeding in patients with cirrhosis [9]. The potential impact of thrombocytopenia on procedural bleeding risk remains unclear, with some studies suggesting an increased risk of bleeding in patients with platelet counts less than 50 × 10^9^/L [10], while others do not show a relationship [11–13]. Despite frequent abnormalities in conventional coagulation tests, in most patients with cirrhosis thrombin generation is preserved [14].

Up-to-date international clinical practice guidelines acknowledge the limitations of standard haemostatic tests, particularly prothrombin time/INR, in predicting procedure-related bleeding, and as a consequence, no longer recommend routine pre-procedural blood component prophylaxis based on these parameters [15–17]. Despite this, clinical practice has been slow to change, and patients with cirrhosis are still frequently given blood component products, based on their INR and platelet count abnormalities, in an attempt to reduce peri-procedural bleeding [18]. Although the overall risk of bleeding for patients with cirrhosis undergoing procedures remains low, particularly for low-risk procedures [19–22], there is a subset of patients who do bleed, which is likely to explain the clinician tendency to “correct” standard coagulation tests abnormalities with pre-procedural blood product administration that has continued regardless of guidelines recommending against it. This is despite an acknowledgement that there are significant risks associated with blood component therapy, particularly large-volume products such as FFP, including fluid overload, pulmonary oedema, allergic reactions, and elevation in portal pressure [23, 24].

It is imperative that an improved method of assessing procedure-related bleeding risk in patients with cirrhosis is developed and validated, so that blood products can be efficiently and effectively allocated in this patient population.

In recent years, ‘global’ coagulation tests, such as ThromboElastoGraphy(TEG) and Rotational Thromboelastometry (ROTEM) have been developed. Whereas platelet counts, INR and APTT measure only individual components of the haemostatic system, ROTEM and TEG measure the viscoelastic properties of whole blood to provide a measure of overall haemostasis, from clot formation to clot retraction and fibrinolysis [25]. These point-of-care tests provide quick results and have been used to assess coagulation and guide blood product transfusion in a number of surgical and trauma settings, including liver transplantation [26]. In these settings, these tests predict bleeding and thrombotic risks more accurately than conventional coagulation tests, are associated with a reduction in intra-operative blood loss, decreased rates of blood product usage, and improved mortality [27].

Recently, there has been increasing interest in the use of these viscoelastic tests in liver disease outside of the transplant setting. Two randomised controlled trials have found that using TEG to guide prophylactic blood component transfusion in patients with cirrhosis undergoing invasive procedures resulted in decreased blood product use without affecting bleeding outcomes [28, 29]. Similarly, a randomised controlled trial found that using ROTEM to guide pre-procedural blood product transfusion in the cirrhotic *children* having invasive procedures led to decreased FFP and platelet use, with no significant difference in procedure-related bleeding [30].

We designed this study to assess the utility of a ROTEM-based algorithm to guide prophylactic blood component delivery in *adult* patients with cirrhosis undergoing invasive procedures. We hypothesise that ROTEM-based decision-making will lead to a reduction in pre-procedural blood component usage, particularly FFP, compared with standard of care. We anticipate that there will be no difference in bleeding complications between the two groups.

By attempting to establish the role of ROTEM in assessing coagulation status in patients with chronic liver disease, we hope to improve the identification of those patients with cirrhosis who are and are not at increased bleeding risk, and in turn, improve the identification of those patients who are most likely to benefit from blood component transfusion.

### Objectives [31]

The primary efficacy aim of this study is to compare the proportion of procedures requiring prophylactic transfusion with blood components between ROTEM-based decision-making and standard of care. The primary safety aim is to compare procedure-related bleeding between ROTEM-based decision-making and standard of care management. The secondary objectives of the trial are to compare the following between ROTEM-based decision-making and standard of care: (1) the number of FFP, platelet and cryoprecipitate transfusions given as bleeding-prophylaxis; (2) the occurrence of procedure-related non-bleeding complications (thromboembolism, infection), length of hospital stay and survival; and (3) transfusion-related events.

### Trial design {8}

The RECIPE trial is a randomised controlled trial with a 1:1 allocation to one of two parallel groups (pre-procedural blood component prophylaxis based on a ROTEM algorithm or based on conventional coagulation tests as per standard of care). It is designed as a superiority trial aiming to demonstrate that a ROTEM-based algorithm will lead to a significant reduction in prophylactic blood component usage compared with standard of care. An attempt to blind the patient and proceduralist to the allocated intervention arm will be made. All participants will have otherwise standard peri-procedural care.

## Methods: participants, interventions and outcomes

### Study setting {9}

Participants will be recruited from the inpatient wards and outpatient clinics at several tertiary hospitals and liver-transplant centres across Australia. A list of up-to-date study sites can be obtained through the Australian and New Zealand clinical trials registry.

### Eligibility criteria {10}

#### Inclusion criteria: participants

To be eligible for randomisation, participants must meet the following criteria:

- Males and females aged 18 years or older
- Have liver cirrhosis (of any aetiology), which is biopsy proven or defined by any two or more of the following: Fibroscan reading > 12.5 kPa, APRI (AST to platelet ratio index) score $\ge$ 2, ultrasound features of cirrhosis, evidence of portal hypertension on imaging and/or presence of varices, compatible clinical features.
- Planned for an invasive procedure
- Coagulopathic based on conventional coagulation tests and considered for pre-procedural blood component prophylaxis as per hospital-specific standard of care
- Able and willing to provide informed consent
- Able to speak and understand English

#### Inclusion criteria: invasive procedures

Any procedure (excluding procedures involving the central nervous system) for which an eligible patient would normally be considered for blood component prophylaxis prior to the procedure will be included.

Procedures will be classified into low-risk and high-risk, based on the likelihood of bleeding and the clinical significance of any related bleeding. Classification will be based on that used published in the American Gastroenterology Association guidelines [32]. Low-risk procedures will account for no greater than 25–30% of all procedures.

#### Exclusion criteria

Candidates who meet any of the following criteria are excluded from randomisation:

- Coagulation disorders (other than those relating to liver disease)
- Patients on anticoagulant medications (e.g. warfarin, enoxaparin, rivaroxaban, dabigatran, apixaban).
- Patients on anti-platelet aggregation agents other than aspirin (e.g. clopidogrel, ticagrelor)
- Patients in whom it is difficult to obtain blood samples due to venous access difficulties
- Active malignancy (EXCEPT; hepatocellular carcinoma, cervical carcinoma in situ, treated basal cell or squamous cell skin carcinoma, superficial bladder tumours [Ta, Tis & T1] or any cancer curatively treated >3 years prior to study entry)
- Patients who have received FFP, platelet transfusion, cryoprecipitate in the week prior
- Patients with stage 4 or 5 chronic kidney disease
- Patients receiving renal replacement therapy
- Patients with active sepsis

### Who will take informed consent? {26a}

The trial will be explained and informed consent will be obtained by study investigators at the individual study sites. In addition to a verbal explanation, potential participants will be provided with a written patient information and consent form (PICF) and adequate time to read this. The participants will have the opportunity to ask questions.

### Additional consent provisions for collection and use of participant data and biological specimens {26b}

As part of the standard consent form, participants will also be consented for additional blood samples to be stored and used for further coagulation testing in the future.

## Interventions

### Explanation for the choice of comparators {6b}

The RECIPE trial is designed to compare ROTEM-guided blood component prophylaxis with standard of care blood component prophylaxis in patients with cirrhosis having invasive procedures.

At the time of our protocol development, there had been no previously published ROTEM decision-making algorithm for patients with cirrhosis having invasive procedures. The ROTEM-based algorithm we have developed is based on those that have been published for use in other settings (liver transplant and cardiothoracic surgery [26]) and then modified by an expert panel of haematologists (co-investigators HN and CH).

For our standard of care arm, the INR and platelet cut-offs used to guide blood component prophylaxis have not specifically been defined and instead will be at the discretion of the individual study sites and proceduralists. This decision was made on the basis of previous work published by our team which found significant heterogeneity in routine prophylactic transfusion practices across Australian institutions for different procedures and different trigger INR and platelet cut-offs [18].

### Intervention description {11a}

All participants will have baseline pathology including standard coagulation testing and a ROTEM analysis.

ROTEM analysis will be performed by one of the investigators using the ROTEM-sigma device located at the study site, within 4 h of the participant’s blood draw. The ROTEM-sigma performs all analyses on citrated whole blood and will be run according to the manufacturer’s instructions. Each blood sample will be tested using four ROTEM assays with different reagents provided by the manufacturer: each of these assays evaluates overall clot formation and strength. The EXTEM evaluates clot formation mimicking the conventional extrinsic pathway, INTEM evaluates clot formation mimicking the conventional intrinsic pathway, FIBTEM evaluates the contribution of fibrinogen to clot formation, and APTEM which evaluates any antifibrinolytic effect. All other laboratory tests will be run by the Hospital pathology laboratories at the study sites.

#### Standard of care intervention

Participants assigned to the standard of care intervention will receive prophylactic blood components (FFP, platelet transfusion, cryoprecipitate) based on INR and platelet count prior to their procedure. The INR and platelet cut-offs used to determine whether blood product is required, the type and dose, will be based on standard of care at the hospital where the procedure is taking place, and as such may vary between sites and procedures. The required blood products are ordered and administered to the patient immediately prior to the procedure.

#### ROTEM-based intervention

**Fig. 1:**
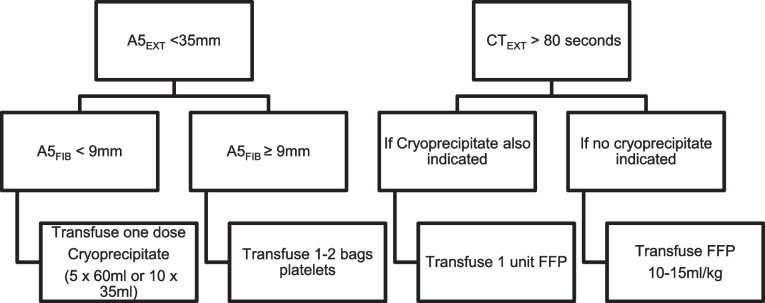
ROTEM algorithm for prophylactic blood component

For participants assigned to the ROTEM-based decision-making arm, the following ROTEM parameters will be used to determine whether pre-procedural blood product should be given (see Fig. 1 below):

- Clotting time using EXTEM (CT_EX_)
- Amplitude at 5 min using EXTEM (A5_EX_)
- Amplitude at 5 min using FIBTEM (A5_FIB_)

If it is determined that blood products are required, these are ordered and administered to the patient immediately prior to the procedure.

Patients in both the ROTEM and standard of care arms will have the same peri-operative management aside from that related to the method used to assign prophylactic blood component.

### Criteria for discontinuing or modifying allocated interventions {11b}

All participants can decide to withdraw from the study for any reason and at any time during the study. If it is before the time of intervention, prophylaxis will be guided by standard of care. The investigator can also decide to withdraw a patient from the study if he/she considers it as necessary (e.g. non-respect of at least one of the selection criteria known after inclusion, or for patient safety). When a patient withdraws from the study, all collected data up until the time of withdrawal will be analysed unless the participant requests deletion of their data.

### Strategies to improve adherence to interventions {11c}

A note stating that the patient is enrolled in RECIPE will be written in the participant’s electronic medical records, with a specific statement to contact the treating team prior to the administration of any blood component leading up to the procedure, outside of the emergency setting.

### Relevant concomitant care permitted or prohibited during the trial {11d}

Participants who receive the following blood components in the seven days prior to the procedure (aside from what is allocated according to RECIPE protocol) will be excluded from the trial (FFP, platelet transfusion, cryoprecipitate, Prothrombinex®). During and post-procedure, any blood components or additional procedures required are permitted and will be recorded.

### Provisions for post-trial care {30}

Participants are encouraged to seek assessment and/or treatment from their general practitioner or treating team if they suffer from any medical problems during the trial. They are also encouraged to make their study team aware of any new medical issues, so their relationship with the trial can be evaluated.

### Outcomes {12}

The primary efficacy outcome is the difference in the proportion of procedures requiring prophylactic blood component transfusion, defined as the procedures before which any combination of FFP, platelets and/or cryoprecipitate is given to the participant. The primary safety outcome is the difference in procedure-related bleeding complications between the two groups. Bleeding complications will be defined in accordance with the International Society of Thrombosis and Haemostasis guidelines and include clinically significant bleeding with a ≥ 20 g/L drop in haemoglobin or requiring blood transfusion, readmission, or intervention [33].

The secondary outcomes are comparisons in the following between the standard of care and ROTEM-based decision-making arms: amount (in units) of individual prophylactic blood products (FFP, platelets, cryoprecipitate) given; transfusion-related side effects, procedure-related complications other than bleeding (e.g. thromboses, infection, organ damage) assessed at day 0, day 7 and day 28, hospital length of stay (in days), and survival (which will be further delineated into procedure-related or not). Hospital length of stay and survival will be assessed at day 28, which is the end of the trial.

### Participant timeline {13}

We aim to investigate 56 interventions in cirrhotic patients with coagulation abnormalities at the study sites. We will recruit patients from both outpatient clinics and inpatient wards who are scheduled for an upcoming invasive procedure, and who would usually be considered for blood components as bleeding prophylaxis prior.

**Fig. 2:**
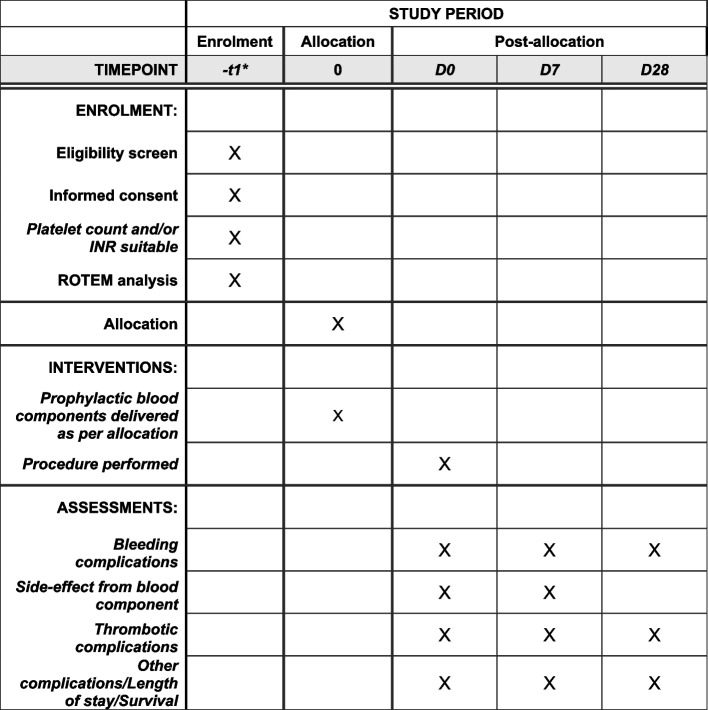
RECIPE schedule of enrolment, interventions, and assessments. *Within 24 h of procedure for inpatients and 72 h of procedure for outpatients

The schedule of enrolment, intervention and assessments is displayed in Fig. 2. All patients meeting eligibility criteria will have a blood sample taken within 24 h of their upcoming planned procedure (72 h for stable outpatients). If platelets and/or INR are within the range that the patient would normally be given FFP or platelet transfusion prior to the procedure as per standard of care (institution-based), ROTEM analysis will be performed, and the patient will proceed to the randomisation phase.

Enrolled patients will be randomised in a 1:1 fashion to either procedure-prophylaxis guided by standard of care or ROTEM-based protocol as follows:

1. Patients in the standard of care arm will receive blood product as per standard of care at the hospital where their procedure is taking place.
2. Patients in the ROTEM arm will receive blood product prophylaxis according to ROTEM cut-offs (see Fig. 1 above).

Data will be collected prior to the procedure, immediately post-procedure, 7 days post-procedure and 28 days post the procedure. Data will be collected from the patient directly via medical history taking and examination, and also from the hospital’s electronic medical records.

### Sample size {14}

Previous studies using other global coagulation assays in patients with cirrhosis undergoing invasive procedures have reported a significant (at least 45%) reduction in the need for prophylactic pre-procedural transfusions [28, 29]. Assuming a modest 30% reduction in pre-procedural prophylactic use of blood products, and with a power of 90% and an alpha of 5%, and accounting for possible drop-outs and missing data of 10%, a total of 56 participants or procedures (28 in each arm) will be needed.

### Recruitment {15}

The coordinating site investigators will present the study at unit meetings at the study sites to encourage recruitment and ensure rotating staff are aware of the study. Regular email correspondence detailing screening, enrolment and study completion numbers at the various study site will be sent to all investigators.

## Assignment of interventions: allocation

### Sequence generation {16a}

Participants will undergo computer-generated randomisation using Study Randomizer [Software Application]. (2017) Available at: https://www.studyrandomizer.com. Allocation will be stratified according to procedural risk (low vs. high bleeding risk), platelet count (greater than or less than 30 × 10^9^/L) and INR (less than or greater than 2.0).

### Concealment mechanism {16b}

Site investigators will contact the coordinating site who will run the computer-generated randomisation and allocate the participant to the randomised allocation. Based on the allocation, the site study investigators will determine the prophylactic blood product (if any) required and liaise with the treating time to see if this is ordered before the procedure. The proceduralist and participant will not be aware of the participant’s allocation.

### Implementation {16c}

The site investigators at the individual sites will enrol participants. Once enrolment is complete, the coordinating site will run the computer-generated randomisation and allocate the participant to an intervention arm.

## Assignment of interventions: blinding

### Who will be blinded {17a}

Participants will be blinded to the results of their ROTEM analysis and will not be told whether they are assigned to the ROTEM-based arm or standard of care. However, it is possible that those participants who end up not requiring prophylactic blood product transfusion may deduce that they have been randomised to ROTEM-based decision-making. The proceduralist will also be blinded to the allocation. The study investigator and treating team are not able to be blinded, as it is necessary for these staff to order and administer the blood products. The person performing the randomisation will be blinded to the result of the ROTEM analysis. Given the hard outcomes of the study, we do not think the lack of blinding of the treating clinicians will greatly bias the results.

### Procedure for unblinding if needed {17b}

The chief investigator can be contacted for unblinding if necessary for patient safety.

## Data collection and management

### Plans for assessment and collection of outcomes {18a}

Baseline data will be collected from participant interviews or the electronic medical records by study site investigators at the individual study site and inputted directly into the REDCAP database. The assessment of outcomes will be in accordance with the pre-defined definitions of the outcomes such as bleeding, SAEs etc. and conducted by the Steering Committee overseeing the trial conduct. Study site investigators are all hospital doctors who have specialist expertise in the management of patients with advanced liver disease. The investigators performing the ROTEM analysis will receive formal training on how to operate a ROTEM sigma device and basic interpretation of the ROTEM analysis. by Werfen Australia.

### Plans to promote participant retention and complete follow-up {18b}

The RECIPE study follow-up duration is only 28 days. For participants who are discharged from the hospital before the day 7 or day 28 follow-up assessments, these will be conducted over the telephone to maximise participant retention and follow-up. For participants who deviate from intervention protocols, an attempt will still be made to collect all outcome data.

### Data management {19}

Data will be entered by the study investigators directly into a REDCap (Research Electronic Data Capture) database hosted by Alfred Health. REDCap is a secure, web-based software platform designed to support data capture for research studies [34, 35]. Data from all participants will be entered using the participant number rather than the participant name. The data will be stored on REDCap for 10 years following the completion of the study. Data will not be stored elsewhere. NJ and SKR at the coordinating site will have access to the data. No other investigators will have access to the data, however, the compiled results will be disseminated to the other investigators for review prior to publication.

### Confidentiality {27}

All collected information concerning participants in this study will be treated as confidential and securely stored study site or using secure electronic platforms. All paper-based participant information is stored in locked filing cabinets in research facilities with limited access. All data collection, process, and administrative forms are identified by a coded participant identification number to maintain participant confidentiality. All records that contain names or other personal identifiers, such as informed consent forms, are stored separately from study records identified by code number. Data will only be disclosed with permission of the individual participant, or in compliance with the law. Access rights, as provided by the law available in each participating state, can be exerted at any time by all the participating patients.

### Plans for collection, laboratory evaluation and storage of biological specimens for genetic or molecular analysis in this trial/future use {33}

There are no plans for any future genetic or molecular analysis of biological specimens.

## Statistical methods

### Statistical methods for primary and secondary outcomes {20a}

All statistical analyses will be performed using STATA.

The primary efficacy outcome will be an intention-to-treat analysis, comparing the proportion of procedures requiring prophylactic blood component transfusion between the two randomised groups. The primary safety outcome will be a per-protocol analysis comparing the percentage of procedures associated with a procedure-related bleeding complication between the two groups. The secondary outcomes will either be analysed as intention-to-treat analyses (for survival), modified intention-to-treat (comparing specific blood products or transfusion-related side effects) and per protocol analyses (for procedure-related complications other than bleeding and hospital length of stay). The primary outcomes and secondary outcomes comparing proportions or percentages will be compared using chi-squared or Fisher’s exact tests. The secondary outcomes examining continuous variables will be compared using the Mann–Whitney test. A *p*-value less than 0.05 is considered statistically significant in a two-sided test.

### Interim analyses {21b}

No interim analyses are planned.

### Methods for additional analyses (e.g. subgroup analyses) {20b}

There are no additional subgroup analyses planned at this stage. Given the RECIPE trial is an RCT, we are not adjusting for any variables.

### Methods in analysis to handle protocol non-adherence and any statistical methods to handle missing data {20c}

The methods used to analyse protocol deviations will depend on the stage at which the protocol deviation occurred. Due to the limited data collection points, we do not anticipate missing data.

### Plans to give access to the full protocol, participant-level data and statistical code {31c}

We will make data, analytic methods, and study materials available to other researchers upon request subject to the intent and purpose of the request particularly in relation to competing additional study hypotheses and planned analyses.

## Oversight and monitoring

### Composition of the coordinating centre and trial steering committee {5d}

The RECIPE trial steering committee is composed of the principal investigators and a subset of co-investigators, primarily based at the coordinating centre. The steering committee directs all aspects of the study, including protocol design, writing of the patient information and consent form, set-up and design of data collection database, monitoring of study progress and quality, and resolution of issues that arise during follow-up. Independent monitoring of study safety and quality is also provided by a DSMB (see below).

### Composition of the data monitoring committee, its role and reporting structure {21a}

An independent data safety monitoring board (DSMB) will be set up to monitor the conduct of the study and the safety of the study participants. All members of the DSMB are independent of the investigators and do not otherwise participate in the study or have other conflicts of interest. The DSMB members have experience in the fields of hepatology and/or haematology that is required to provide appropriate oversight for the trial. Any serious adverse events, particularly bleeding, will be reported to the DSMB within 24 h. The DSMB will meet after the recruitment of 15 and 28 participants. At these times, the DSMB will evaluate the timeliness of participant recruitment, adherence to the protocol, and the potential of the study to meet the stated goals; the quality and integrity of the data, participant safety, particularly trends in bleeding events and relationship to the study procedures; and factors external to the study when these may have an impact on the safety of the participants or the ethical conduct of the study. The DSMB will make recommendations, to the investigators on the continuation, termination, or other modifications of the study protocol.

### Adverse event reporting and harms {22}

At each scheduled follow-up, participants will be assessed for adverse events. Additionally, participants are provided with phone numbers to contact the study coordinators if needed in between scheduled visits. Study staff at the individual study sites will report any adverse events to the coordinating study site. AEs and SAEs will be defined as per the Australian National Health and Medical Research Council guidelines [36]. An adverse event (AE) will be defined as any untoward medical occurrence in a clinical trial participant receiving an intervention. A serious adverse event (SAE) will be defined as any AE that (a) results in death, is life-threatening, or places the participant at immediate risk of death from the event as it occurred; (b) requires or prolongs hospitalisation; (c) causes persistent or significant disability or incapacity; or (d) results in congenital anomalies or birth defects. Adverse events will be categorised according to the likelihood of relationship to the study interventions (unrelated, unlikely related, possibly related, probably related, or definitely related). All SAEs and serious protocol breaches will be reported to the DSMB, relevant institutions or health services, and/or the ethics and research governance committee within the time frames required.

### Frequency and plans for auditing trial conduct {23}

The governing ethics review committee may choose to audit the trial at any time. No specific audit is planned.

### Plans for communicating important protocol amendments to relevant parties (e.g. trial participants, ethical committees) {25}

Any protocol modifications will have to be approved by both the DSMB and the ethics and research governance committee prior to implementation. They will be then communicated to the individual site investigators both verbally in the format of a virtual meeting and written in the form of an email with the amended documents explained and attached.

### Dissemination plans {31a}

Trial results will be submitted for publication in a peer-reviewed medical journal, no later than one year after the trial’s completion date. The investigators also intend to communicate the trial results to the wider scientific community through presentations at scientific meetings. If the trial cannot be completed (due to early termination of the trial for safety or other reason) interim results will likely still be submitted for publication.

## Discussion

One of the main practical issues faced by the RECIPE trial is the recruitment of participants. Participants meeting inclusion criteria represent a group of patients with advanced liver disease, usually with associated portal hypertension, that makes them at high baseline risk for surgery, and as such, surgery, outside of the emergency setting is often avoided. In the emergency setting, it can be difficult to recruit patients into the trial, as there is limited time to consent the patient and ensure the treating team/proceduralist are all in agreement with the study protocol. To address this, we have delivered a number of presentations and held a number of meetings to familiarise different specialty departments within the study health services with the RECIPE trial.

## Trial status

Protocol version 4: 25/5/2020. The first participant was recruited on the 26^th^ Feb 2021. It is anticipated that recruitment will be completed by Dec 31^st^ 2023.

## Data Availability

Only study investigators from the primary study site will have access to the final trial dataset.

## Abbreviations

AE: Adverse event
APRI: AST to platelet ratio index
DSMB: Data safety monitoring board
INR: International normalised ratio
FFP: Fresh frozen plasma
NBA: National blood authority
PT: Prothrombin time
ROTEM: Rotational Thromboelastometry
SAE: Serious adverse event
TEG: Thromboelastography
